# A Pilot Acceptability Study of an ‘AllPlay Pre-Learn’ Day Program to Facilitate Participation in Organised Physical Activity for Children with Disabilities

**DOI:** 10.3390/ijerph16245058

**Published:** 2019-12-11

**Authors:** Katherine Howells, Carmel Sivaratnam, Tamara May, Ebony Lindor, Nicole Rinehart

**Affiliations:** 1 School of Psychology, Faculty of Health, Deakin Child Study Centre, Deakin University, Geelong 3220, Australianicole.rinehart@deakin.edu.au (N.R.); 2 Murdoch Childrens Research Institute, Parkville, Australia and Department of Paediatrics, Monash University, Clayton 3052, Australia

**Keywords:** disability, organised physical activity, sports participation, football

## Abstract

In a mixed-methods design, the current study aimed to evaluate the acceptability of a junior Australian rules football program across two ‘AllPlay Pre-Learn’ days for children aged 5–11 years with disabilities, based on parent and child responses. Three online surveys were created by health professionals based on existing participation models. Surveys were completed by parents immediately before (*n* = 23), after the ‘Pre-Learn’ days (*n* = 15) and following the conclusion of the community version of the program (*n* = 13). Quantitative findings indicated significant improvements in child ratings around enjoyment of the sport. Qualitative analyses generated three themes around enjoyment in a low-stress environment; the education provided around the sport for parents/children; and, contemplation about playing the football program within their community. Four families (22% of the original attendees) went on to play the sport within a community setting. Despite acknowledged limitations, this study demonstrates preliminary evidence in support of an ‘AllPlay Pre-Learn’ day as a stepping stone to facilitate later participation in a football program within a child’s community. Increased participation would allow children to experience the benefits associated with sport participation, such as motor and social skill development.

## 1. Introduction

Recent research has highlighted the importance of novel interventions that may overcome the barriers of current models of treatment (e.g., interventions held in clinics or health care settings which may be difficult for all families to reach) [1]. It has been said that research examining programs outside the clinic and in more naturalistic settings have the potential to benefit children with neurodevelopmental disorders, as well as their families [2]. Examples of such programs include more lifestyle-based programs such as exercise or physical activity (PA) [1,2]. Current research indicates lower levels of physical activity in children with disabilities during adolescence in comparison to their typically developing (TD) peers [3,4]. In the case of autism spectrum disorder (ASD) however, this changes during the younger years (4–7-year-olds) whereby no significant differences have been found in PA levels [5]. This indicates younger age groups provide a critical window of opportunity to promote good health outcomes through participation in PA programs. PA’s effectiveness has been demonstrated in children with physical disabilities such as cerebral palsy (CP) [6], genetic conditions such as Down syndrome (DS) [7] and neurodevelopmental conditions such as autism spectrum disorder (ASD) [8]. Benefits have been shown to span motoric [9], and aspects of cognitive [10] and socio-emotional domains [11] among disability populations. There has been particular interest in organised physical activities (OPA) (community-based activities) for children with ASD who experience social, communication and motor impairments [12] that often impact on success in group-based activities. Community-based programs themselves are said to be influential in both clinical and public health settings [13]. Our recent meta-analysis of group-based OPA participation indicated benefits to social functioning in children with ASD [14]. Notwithstanding the positive findings, literature has consistently highlighted the barriers which exist to physical activity participation for children with disabilities, e.g., anxiety and confidence levels (described in further detail below). Before children are able to participate meaningfully in sporting programs that occur within their community (e.g., school and club sporting settings), there is a critical need for research into strategies that can reduce the barriers which currently exist.

### 1.1. Preference and Skill-Related Barriers

Qualitative research indicates a common barrier to physical activity participation is a lack of enjoyment or boredom among children with disabilities such as ASD [15] and genetic conditions such as Down syndrome [16]. Preference for other non-physical activities has also been noted as a barrier, with Obrusnikova and Cavalier [15] indicating that 94% of their child sample reported engagement in technologically based activities as the reason for not engaging in physical activity after school.

Lower confidence levels in physical skills have also been noted as a barrier to physical activity participation in children with both neurodevelopmental and physical disabilities [17]. Parents in this qualitative study indicated their children felt a loss of confidence when comparing their skills with (TD) peers [17]. A lack of skills itself has also been consistently identified as a barrier to physical activity participation for children with a range of disabilities [15,16,17,18] and as a barrier to team-based sporting specifically [19]. Research involving children with CP indicated that even if a child is interested in a particular physical activity, the belief that the activity is too difficult hinders participation [20], a finding also cited in research with ASD child populations [15]. Participation can also be hampered by coaches or trainers with inadequate skills/training to adapt the activity and cater to the skill levels of children with disabilities [21].

### 1.2. Anxiety Levels

For children with disabilities, unfamiliarity with the environment can also act as a hindrance in participation [18] and may be a trigger for anxiety, with this anxiety also posing as a barrier to participation [22]. Specifically, children with ASD often have difficulties adjusting to novel or unfamiliar situations [12] which may lead to them feeling overwhelmed [22] and heighten anxiety levels, possibly contributing to a lack of participation in activities these children may have otherwise enjoyed. Furthermore, individuals with ASD can often have ‘hypo or hypersensitivities’ to sensory aspects of their environment (e.g., sounds and textures) [12]. More recent research indicates children with CP also experience ‘concomitant sensory impairments’ [23]. The sensory characteristics of an environment such as touching mud or getting wet [19] are thought to impede participation in activities such as organised sport [24]. Parental concerns and anxieties around how their child may perform or behave can also prevent parents from enrolling their child in sporting activities [18]. One qualitative study indicated parents of children with CP often fear to observe their child struggle (i.e., losing) [20].

### 1.3. Cost and Time Related Constraints

Whilst literature indicates that community-based programs more generally can be considered cost effective [25], Shields, Synnot and Barr [18]’s systematic review identified the costs associated with travel, equipment needed and facilities as a barrier to PA participation for children with disabilities. Cost has also been identified as a key barrier within more recent disability [26,27] and TD literature [28]. In addition to financial burdens, time constraints have also been consistently identified as barriers to participation. Specifically, qualitative research examining parents of children with ASD indicated they often do not have the time to support PA participation for their children [27]. Clinicians in a recent qualitative study concerning children and youth with physical disabilities indicated that therapy related commitments alongside day to day activities may influence the time constraints of this population when it comes to PA participation [26]. In light of these findings, there is a need for a cost-effective program which allows exposure to a PA whilst offering low time commitments, to enable greater participation for children with disabilities.

### 1.4. General Strategies to Overcome Barriers

In clinical settings, the use of psychological techniques such as graded-exposure may be effective in reducing anxiety levels in individuals with developmental disorders [29]. These techniques involve exposing one to previously avoided situations or fears in smaller stages or steps [30]. Such techniques may involve exposing the individual to a certain situation for a fraction of the time, in a less threatening manner, and can involve using tools such as visual modalities to support learning.

Graded exposure techniques have been used successfully across a range of settings, for example, to increase food repertoires [31] and to facilitate the transition into new environments such as school [32]. A previous case study suggested that when paired with positive reinforcement, gradually exposing a child with ASD to previously avoided rooms in a school setting (music, gymnasiums and gross-motor exercise rooms) not only increased the child’s tolerance of those rooms but also increased the child’s participation in activities in these rooms without problem behaviours [33]. These exposure strategies, therefore, present themselves as opportunities to reduce some of the barriers associated with sport participation for children with disabilities.

### 1.5. A Potential Strategy within the Sporting Setting

It is recognised that it is not always feasible for psychologists and health professionals support children within the community. Kazdin [1] summarises the idea of ‘task-shifting’, whereby the ‘lay’ people administer, for example, psychological techniques within community-based settings [1]. Another idea for expanding the reach of interventions is through ‘disruptive innovations’ which centers on the idea of bringing programs to the individual, rather than the reverse [1]. Within a sporting setting, the adoption of these principles could include the training of coaches and volunteers to adapt sessions to suit the child (an area of which is recognised as vital [34]), using psychological principles. Moreover, given the time and financial barriers that exist to participation, it is important that programs aimed at reducing barriers also consider this burden on families. The current study examines a cost-effective, brief intervention (single day and free of charge) which incorporates the psychological technique of exposure within the context of Australia’s most popular national junior football program [35] known as NAB Australian Football League (AFL) Auskick (referred to as Auskick hereafter). This program incorporates some of the principles outlined in Kazdin [1] and was developed around the idea of ‘pre-learning’. Through ‘pre-learning’ children are provided with opportunities to develop an interest and skills in activities before ‘learning’ the material [36]. In this context, children have an opportunity to develop an interest and familiarity with basic skills in a peer environment, before engaging in/paying for the program within their communities. It may also allow children and their parents to see if they enjoy the sport before committing to a full season. This is important given research has identified safe, learning, social and family contexts as important to enable PA participation [37]. To the best of our knowledge, this is the first paper to assess this type of brief program in a physical activity setting for children with a range of disabilities.

In a recent study, parents of children with ASD indicated that one of the benefits of playing the OPA Auskick program is that it expanded their child’s interests, mentioning that while there are barriers to participation, joining in with the adapted programs can help address them whilst improving parent-reported motor domains such as soccer kicking and object control skills [19]. It is possible that exposing children with disabilities to the sport, through an ‘AllPlay Pre-Learn’ day (referred to as ‘Pre-Learn’ hereon after) that is carefully tailored to support participation and inclusion (without cost and time related commitments), may increase interest in the sport and decrease child anxiety levels. This study examined the ‘acceptability aspect of feasibility designs’, allowing us to assess how the recipients of the program reacted to it [38]. Acceptability, as described in Harrison, et al. [39], can involve several elements including the recipients viewing the program as suitable (favourable) and effective. We aimed to understand ‘acceptability’ through assessing whether the ‘Pre-Learn’ program could (1) reduce common barriers which may impact of participation in the community Auskick program (e.g., enjoyment related factors, and anxiety); (2) be useful to families as indicated through parent feedback, and (3) result in progression to attending the program within the child’s community.

## 2. Materials and Methods

### 2.1. Participants

Children with disabilities were recruited for two separate ‘Pre-Learn’ days from a range of methods. Advertisement flyers were distributed to various early intervention services located throughout Victoria, Australia (i.e., ABA therapy clinics, physiotherapists, psychology clinics, etc.). Social media was also utilised via the Facebook pages of several early intervention services, the research institution webpage, and several partnering AFL football clubs. The first day took place in March 2018, and the second in April 2018.

Twenty-eight children were registered to participate across the two ‘Pre-Learn’ days. Eighteen children with disabilities in total attended across the two days (*n* = 6 on day one; *n* = 12 on day two) (15 boys; 3 girls). One parent or caregiver per child completed the survey component of this study. For inclusion in the evaluation aspect of this study, children must (a) have had a parent-reported disability, (b) and had not participated in previous seasons of the community football game. If a child attended but did not actively participate in the session parent responses were still examined as part of the qualitative component.

**Figure 1:**
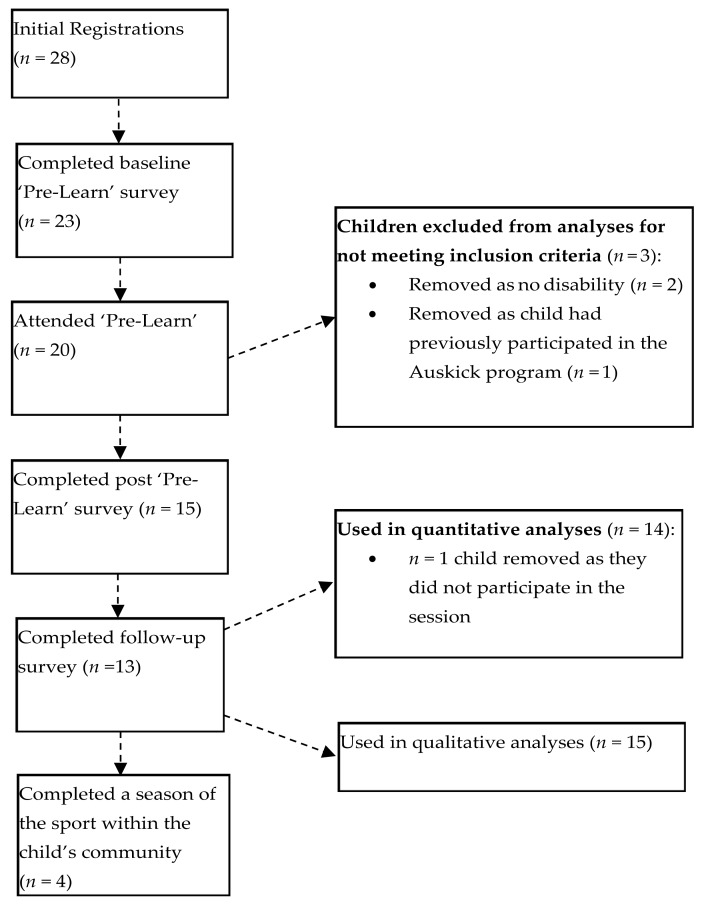
Participation rates in each aspect of the Auskick ‘Pre-Learn’ day evaluation.

Fifteen parents met the above inclusion criteria and completed both pre-and-post ‘Pre-Learn’ day surveys (responses from participants who met inclusion criteria: *n* = 2), hence 15 children were able to be included in final analyses (see Figure 1 for registration and participation rates). The fifteen children (12 boys; 3 girls) were aged between 5–11 years (*M* = 8.07; *SD* = 2.19) and participated in one of the two days (*n* = 5 on the first day; *n* = 10 on the second day). Ten children had a parent-reported diagnosis of ASD, 3 had a diagnosis of CP, one had a diagnosis of Down syndrome and one had a combined diagnosis of ASD and CP. Of the children with a diagnosis of CP, *n* = 2 were considered a gross motor function classification system (GMFCS) level of I, and **n* = 2* were considered GMFCS III (both requiring four-wheeled walkers for mobility support) (see Table 1 for additional demographic information).

### 2.2. Program: An ‘AllPlay (AllPlay Is a Registered Trademark) Pre-Learn’ Day

Both ‘Pre-Learn’ days ran for 60 min; the first day was aimed at children with physical disabilities and was run in an outdoor setting, whereas the second was aimed at children with neurodevelopmental disabilities and was conducted indoors on a basketball court due to weather conditions. The Auskick program specifically is based on the sport Australian rules football [40]. The aim is to score as many points as possible through kicking an ovoid-shaped ball through sets of four equally spaced posts. Players move the ball towards their goals via kicking, bouncing the ball while running, or handballing [40]. Both days closely followed the structure of a typical session in the community junior program starting with group warm-up games and activities. Children then broke into age-related groups to practice various football skills such as handballing and kicking to other children, volunteers or towards a target, marking (catching) and bouncing skill practice. A ‘sensory room’ was available for children to opt in and out of if they were feeling overwhelmed or anxious by the activities. The sensory room contained beanbags, calm music, dim lighting, drawing utensils and ‘social stories’ on the walls pertaining to playing Auskick, anxiety or fear of the game and the environment. A recent meta-analysis found that visual over verbal instructions were important to maximise motor related outcomes [41] and this was adopted into the current study via the use of visual tools to explain activities (during the second day) and through social stories. Using visual techniques is said to enhance the meaning of communication [42]. Children were able to take breaks from the activities as often as was needed for the child. The need for the use of the sensory room was determined by parents.

### 2.3. Coach Training and Demographical Information

Each ‘Pre-Learn’ day was supervised by a head coach and several volunteers. Each coach completed a 1-h training session prior to the day outlining strategies for coaching children with disabilities. The training briefed coaches on how the day would be run and what activities would be carried out. The strategies taught during the coach training sessions included evidence-based inclusive teaching practices based on strategies outlined on the AllPlay Footy coach resource page [43]. Research has highlighted the importance of modifying activities to facilitate successful participation [17,44] and has shown to be efficacious in a previous adapted Auskick program [19]. There were two training sessions run, the first before the initial ‘Pre-Learn’ day aimed at children with physical disabilities, and the second before the program aimed at children with neurodevelopmental disabilities. Both training sessions highlighted strategies that could be used to accommodate children with different disabilities. The training included techniques for making the game more inclusive, derived from principles of the CHANGE IT (coaching, how to score, area, number of players, game rules, equipment, inclusion and time) approach (see AllPlay Footy [45] for further explanations), which provides guidance around how to modify the game to suit each child. The CHANGE IT approach has been used previously by Sports Australia’s initiative ‘Playing for Life’ [46]. Each training session went through a number of scenarios and a group discussion around how to modify the game using CHANGE IT principles (Table 2 presents an example used in each session and how coaches could modify the game).

Of the seven coaches, five reported experience levels. One had coached for 2 years, two had coached for 3 years, one for 4 years and one had coached for 5 years. Three of these five coaches had previous experience working with children with disabilities (20 years: *n* = 1; 4 years *n* = 2). Feedback from the coaches following the training and the ‘Pre-Learn’ days indicated 100% (*n* = 7) coaches strongly agreed that the training sessions were ‘easy to understand’ and 57% (*n* = 4) coaches strongly agreed with the statement “the training provided enough information about my role at the ‘AllPlay Pre-Learn’ day” (the remainder *n* = 3 coaches somewhat agreed). Furthermore, of the *n* = 5 coaches who responded to the question, “How useful was what you covered in the coaches training session for the ‘AllPlay Pre-Learn’ day”, 80% (*n* = 4) indicated it was extremely useful and 20% (*n* = 1) indicated it was very useful. During the ‘Pre-Learn’ sessions, visual inspection by researchers indicated coaches implemented some of the suggestions from the training sessions, e.g., allowing for the ball to be kicked off either the ground or a cone and the use of visual modalities such as pictures to help children understand activities. No rigorous fidelity checking was undertaken, however.

### 2.4. Procedure

Ethical approval was obtained through the Deakin University Human Research Ethics Committee (DURHEC). The ethics identification code for this project is 2017-365 and this was approved on the 6th December, 2017. Following ethical approval, recruitment flyers were distributed to the abovementioned services. In accordance with the Declaration of Helsinki, all registering parents provided written informed consent, whereas children were required to give verbal assent to participation during the ‘Pre-Learn’ days. Parents were then required to complete the baseline survey online prior to each ‘Pre-Learn’ day. The post online survey was then completed within four weeks following each event (except for one participant who completed it five weeks after the event). A follow-up online parent survey was then completed following the conclusion of the official Auskick season (around September 2018 however this differs depending on the specific Auskick centre) with the average time between post and follow-up survey completion being 18 weeks (SD = 3.09) (see Figure 1). As mentioned, coaches were provided with a training session specific to coaching Auskick for children with disabilities prior to the ‘Pre-Learn’ days.

### 2.5. Measures

#### Online Surveys

Three online surveys were developed for the purposes of this evaluation using Qualtrics survey software. The baseline ‘Pre-Learn’ survey contained basic demographic information (i.e., child’s date of birth, gender, the location which they attended the ‘Pre-Learn’ day, diagnosis and therapies received), information on previous participation in the Auskick program and general organised physical activity. A series of Likert-scale style questions were also presented to parents. The overall stem of this question was as follows “How would you rate your child in the following areas in relation to Auskick?” The items presented aimed to ascertain children’s skill levels and enjoyment of Auskick (see Table 3). Answers could range from “a great deal” to “none at all”. Each child was also asked in the survey to rate how much they “like Auskick”, on a Likert-scale ranging from 1–5 with 5 being “love it” and 1 being “really don’t like it”. These surveys were originally developed by a team of health professionals including clinical psychologists and a physiotherapist. Questions were formulated based on the participation model proposed by Imms, et al. [47] which indicates participation involves attendance and involvement, which comprises components of competence, confidence and satisfaction related domains, preferences for activities as well as environmental influences.

The post ‘Pre-Learn’ survey contained the same nine Likert-scale questions. This survey also contained several questions regarding how enjoyable the day was for the individual completing the survey and their child (answers could range from “extremely enjoyable” to “not at all enjoyable”). The option of providing qualitative, open-ended feedback was also provided. Parents/caregivers were also asked to rate how useful the day was for them and their child (from “extremely useful” to “not at all useful”) and offered an opportunity to provide open-ended feedback about this question and the day (see Table 4). Like the initial survey, children were asked to rate their enjoyment of Auskick again (from “love it” to “really don’t like it”) and were also asked to rate their enjoyment of the day.

The follow-up ‘Pre-Learn’ survey contained questions around whether the child went on to complete a season of the sport within the child’s community, and how long they participated. Parents were again asked to rate the same nine areas (see Table 2). Parents/caregivers were again asked to provide qualitative feedback around whether they would recommend the ‘Pre-Learn’ day to families of children with additional needs. Children were also asked to rate how much they “like Auskick”.

### 2.6. Data Analysis

Quantitative data were analysed using IBM SPSS Statistics Version 25. As all quantitative variables were ordinal, Wilcoxon signed rank tests were used to assess change between pre-and-post ‘Pre-Learn’ surveys. The median was reported over means and standard deviations as this is more appropriate in non-parametric analyses [48]. Parents were not excluded from overall analyses if data were missing on one variable, hence there are smaller numbers in one variable used in quantitative analyses (specifically enjoyment of Auskick). While *n* = 13, parents completed the follow-up survey, questions pertaining specifically to Auskick were only presented to those who enrolled their child in Auskick. There was not enough data to assess changes across the three-time points for most variables. The exception to this was child ratings of how much they “like Auskick”. Friedman’s ANOVA was used to assess changes in this variable. Significance levels were not adjusted for follow-up analyses, as per recommendations by Saville [49] and O’Keefe [50].

Thematic analyses on qualitative data were conducted using NVivo 12. Themes were identified using an inductive approach, which allowed the authors to create themes that are strongly linked to the data, rather than creating pre-defined themes [51]. Analyses were conducted in accordance with the six phases identified in Braun and Clarke [51]. One rater (author KH) independently generated initial codes from the data, codes were then reviewed by a second-rater (TM). Authors KH and TM then organised codes into broader themes. KH and TM discussed the broader themes and each theme was refined and agreed on by the two raters with a third rater available to resolve any discrepancies. Results were triangulated or discussed together to assess completeness, convergence or dissonance between results [52].

## 3. Results

### 3.1. Quantitative Results

#### 3.1.1. Enjoyment, Anxiety and Skill Level Related Factors

**Figure 2:**
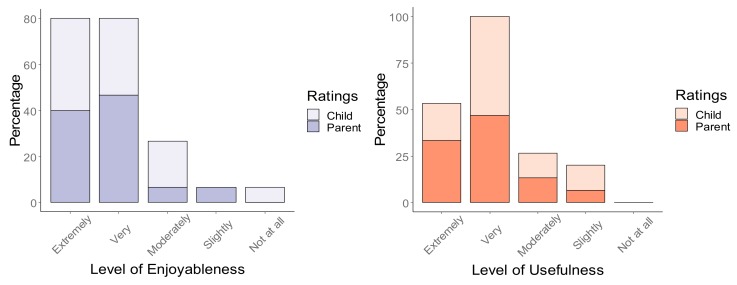
Post parent-reported enjoyment and usefulness (%) for parents and children.

Quantitatively, 86.67% of the 15 parents rated the ‘Pre-Learn’ day program as very enjoyable or above, with 73.33% of the 15 parents rating the day as very enjoyable or above for their children (see Figure 2). Regarding significance testing, the item “level of anxiety in relation to playing Auskick” demonstrated a trend towards significance using the Wilcoxon signed rank test. No other items reached significance (see Table 5 for pre-post median values and accompanying test statistics).

#### 3.1.2. Child Ratings Regarding How Much They “Like Auskick”

Friedman’s ANOVA indicated that child ratings of how much they “like Auskick” significantly improved across the three time points (before the ‘Pre-Learn’ day [baseline], after it [post], and after the Auskick season concluded [follow-up]) X^2^ (2) = 7.14; p = 0.03; *n* = 11.Post hoc analyses were then conducted using separate Wilcoxon signed rank tests. These analyses indicated a trend towards significance in terms of changes in scores between the baseline (Md*n* = 4.00) and the post-survey (Md*n* = 5.00), and the baseline and follow-up survey (Md*n* = 4.00) conducted after the Auskick season concluded (Z = −2.12, p = 0.06, *n* = 11 for both analyses). However, no changes were found between the post and follow-up surveys (Z = 0.00, p = 1.00, *n* = 11).

#### 3.1.3. The Usefulness of the ‘Pre-Learn’ Program and Contemplation about Further Participation

As per Figure 2, 80% of the 15 parents found the day very useful or above, with 73.33% of parents rating very useful or above for their child. Furthermore, at the pre-time point, of the 15 parents who were included in the evaluation, 27% planned to enroll their child in the community version of Auskick (*n* = 1 participant had found a local club to enroll in), and 67% were undecided. At the post time point, 53.33% of respondents said they intended to enrol their child in the program within their community, 33.33% were not planning to, and 13.33% were undecided.

#### 3.1.4. Actioned Participation

Of participants who completed the follow-up survey (*n* = 13 of the 15 involved in pre-post analyses), four families (31%) went on to complete the Auskick season with all four reporting their child completed the full season of the football program within the community (ASD: *n* = 3; CP: *n* = 1 [GMFCS I]; age: M = 8.79; SD = 1.36; gender: 1 male; 3 females). It is important to note that the family who had found a local club already at the pre-time point did not complete the follow-up survey. Furthermore, *n* = 2 families who enrolled in the Auskick program indicated they planned to enroll their child at the pre-time point, and the remaining two participants were undecided. This number was 22% of the original ‘Pre-Learn’ day attendees (18 children with a disability), hence the number of final participation rates could be higher. Of the children who went on to complete the program within their community, none had an additional intellectual disability (ID) based on parent reports. Table 6 presents the median, minimum and maximum ratings of each variable used in pre-post analyses for the participants who went on to complete a full season of the football program within their community (*n* = 4).

### 3.2. Qualitative Results

**Figure 3:**
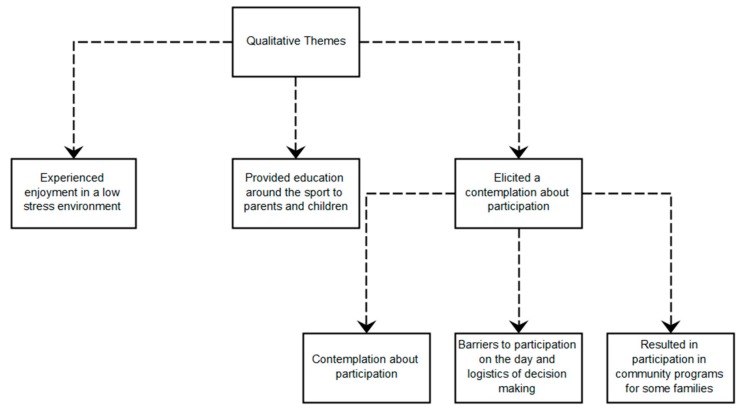
Qualitative themes and sub-themes emerging from open-ended parent survey responses.

In the post-survey, *n* = 13 of the 15 parents provided open ended feedback on behalf of them and their child around the enjoyableness of the day (data missing from *n* = 1 child with ASD and *n* = 1 child with DS). This was the same for the question around usefulness with the exception of parent ratings where data were missing from an additional parent with a child diagnosis of CP. Remainder response rates to questions can be viewed in Table 4. From the responses provided, three key themes emerged from thematic analyses of open-ended survey responses across the post-and-follow-up surveys (see Figure 3). These themes are discussed below:

#### 3.2.1. Theme 1. Experienced Enjoyment in a Low-Stress Environment

This first theme highlighted the enjoyment parents and children experienced following attendance. Specifically, one parent of a 10-year-old girl with ASD noted they “loved seeing [their] kid succeed and have fun, be a good sport and cheer on others, and actually kick a footy!“ Another parent of a five-year-old boy with ASD reported it “reminded [her/him] of how much fun learning footy is and the benefits of physical activity”. Parents also commented that the program allowed children of all abilities to have a go in a non-threatening environment which allowed them to flourish. Parents commented on the “low-stress” and “less daunting” atmosphere the days provided. One parent reported their child experienced elevated anxiety levels on the day, however “…once he was engrossed in the activity, he enjoyed it” (Parent of a six-year-old boy with ASD). The use of a quiet room provided a space to go when children felt overwhelmed, allowing them to later re-join activities, “…After 15 min in the quiet room, he re-joined the activity after watching from the sidelines for a further 15 min” (parent of a five-year-old boy with ASD). Several parents also commented on the enjoyment their child experienced following an opportunity to learn new skills and practice current skills amongst peers of similar skill levels. A parent of a seven-year-old girl with CP (GMFCS I) reported: “It was a great opportunity for children with additional needs to participate and learn some skills with other children with additional needs and not just able-bodied children”. Another parent of an eight-year-old girl with ASD indicated that although they didn’t believe their child understood much around the day “she felt included and didn’t worry when she couldn’t kick the ball and kept dropping it”. A parent of a five-year-old boy with ASD also highlighted the usefulness of the coaches and volunteers in “…helping children like Bob (name anonymised to protect participant identity) to reach their potential” which this parent found to be “truly amazing and a bit emotional”.

#### 3.2.2. Theme 2. Provided Education around the Sport to Parents and Children

The opportunity for children to understand what is involved in the program was another commonly discussed theme, with one parent of a child with ASD mentioning these days are a “…necessary precursor” to participation. Parents articulated that this opportunity also spanned across themselves as a parent, with one parent of a 10-year-old girl with ASD mentioning “getting to see Auskick in practice is always easier than just reading about it”. Furthermore, a parent mentioned they were provided with an opportunity to “test the water for Lisa (Name anonymised to protect participant identity) before enrolling her into Auskick” (parent of a seven-year-old girl with CP [GMFCS I]). This enabled parents to see how interested their child may be in Auskick. More specifically, it provided parents with an “idea of drills available” (parent of a five-year-old boy with ASD), as well as an avenue to see how their child may participate in the drills. Whilst some children enjoyed their experience, there were also children who did not, with one parent of a 10-year-old boy with ASD reporting their child “…clearly articulated that it is not his favourite” which, in turn, influenced parental enjoyment levels. However, it is important to note this parent reported the day as being extremely useful, as they now “know what the activities are and that our son doesn’t enjoy [Auskick]”. This child experienced a co-morbid psychiatric and sensory processing disorder and had only participated in swimming prior to the ‘Pre-Learn’ day.

#### 3.2.3. Theme 3. Elicited a Contemplation about Participation

Several parents reported an intent to join following attendance, reporting that they were currently looking for local clubs to enrol their children in during feedback in the post-survey. Some parents also highlighted their child’s excitement around joining within the community, indicating their child “…can’t wait to start…” (parent of an eight-year-old girl with ASD) and “…would love to play Auskick now” (parent of a seven-year-old girl with CP [GMFCS I]).

##### Barriers to Participation on the Day and Logistics of Decision Making

Parents reported several barriers which influenced participation on the day, and decisions to enrol children in the community program during the post-survey. The follow-up survey also highlighted several logistical issues that influenced parents’ decision making. Regarding barriers, a parent of a six-year-old boy with ASD and CP mentioned sessions contain “too many children and it goes for too long…” with the parent highlighting that “small groups worked best”. Another parent mentioned their child’s “...extreme anxiety prevented him from participating” in ‘Pre-Learn’ (parent of a 10-year-old boy with ASD. Another parent indicated their child’s “behaviour issues and small attention span” was the reason they would not be joining Auskick (parent of an 11-year old boy with DS). Furthermore, age was recorded as a barrier with one parent reporting their child “feels he is too old to join Auskick…” (parent of a 10-year-old boy with CP [GMFCS III]). Several parents reported logistical issues which prevented them from enrolling their child in the community program during the follow-up survey; for example, two parents of boys with ASD reported issues around the day community sessions are held, with one mentioning “logistics [are] difficult as we work on Saturdays” and the other highlighting Friday sessions are difficult as their child is “…too wound up and tired after school…” also mentioning they as parents “…couldn’t find a suitable Sunday session…”. Another parent articulated their child enjoyed the activities he was involved and would like to join, however, the age of the child’s younger sibling (<5) prevented them from enrolling as Auskick has a minimum age requirement of five years.

##### Resulted in Participation in the Community Program for Some Families

Several parents indicated immediately following the program (in the post survey) that they had already enrolled their child in Auskick following attendance at a ‘Pre-Learn’ day. One parent of a seven-year-old boy with ASD highlighted the fact they had now successfully joined their local Auskick club “week three and loving it”. Another parent of an eight-year-old girl with ASD also mentioned they had joined their local club indicating “[they] would never have joined Auskick without this day”.

## 4. Discussion

Given the significant number of barriers that exist to sports participation for children with disabilities and the financial and time-related burden for families, the current pilot study aimed to evaluate a single-day, free ‘Pre-Learn’ program aimed at reducing some of these barriers. The study included children with ranging disabilities and severity levels, which is important when examining programs within the community. Further, this study aimed to evaluate the acceptability aspect of feasibility evaluations regarding two ‘Pre-Learn’ days. This program was tailored for children with disabilities and based on Australian rules football. Specifically, we assessed whether participation could reduce anxiety and improve enjoyment related factors, the usefulness of the program and whether it could result in progression into the community version of the program. Results indicated twenty-two percent (four out of 18) of families went on to complete a season of the sport within their community, inclusive of three children with ASD and one child with CP aged between eight and eleven years. None of the children who went on to participate had parent-reported comorbidities such as anxiety or ID and the child with CP was a GMFCS I. It is important to note that only two parents indicated that non-participation in the community program was a direct result of their child’s behavioural/emotional functioning. Future research should explore similar programs in relation to level of functioning on a larger scale. Furthermore, all the girls who took part in the ‘Pre-Learn’ program (*n* = 3) went on to complete the program in their communities. This is interesting as the rates of males enrolled in the Auskick program specifically are much higher than of females [53]. It may be that participation in a ‘Pre-Learn’ program may also encourage girls to engage in football however as the sample size is very small, further research on the impact of this program on gender needs to be explored. It is also important to note that data were missing for several families. Given one family had indicated they had enrolled in their local Auskick club, it is likely attendance rates would be higher.

### 4.1. Influence on Barriers Such as Enjoyment and Anxiety Related Factors

Results indicated that a high number of parents rated the ‘Pre-Learn’ program as either very enjoyable or above, with parents reporting they also enjoyed watching their child have fun. Many parents also rated the day as either very enjoyable or above on behalf of their children which was supported through child responses which indicated a significant increase around how much the child liked Auskick across the three time points. However, given several participants had co-morbid language and/or intellectual disorders, child responses must be interpreted with caution. It is also important to note that for the participants who completed the full community season, parent-reported ratings indicated their child felt on average ‘a lot’ of satisfaction, pride and enjoyment when playing the sport (these ratings matched those at the post time point), indicating the children who participated in the full program also had positive experiences. Quantitatively, children’s parent-reported skill levels did not increase. This is unsurprising given one 60-min session would not be a large enough dosage to promote motor skill changes. However, qualitative feedback indicated parents felt their children were provided with the space to try new football/motor skills and kick the ball among other children with additional needs. Parents indicated the environment was both non-threatening and low-stress, providing a chance for inclusion in an environment where it didn’t matter if the child couldn’t kick the ball. This, in turn, may minimise anxiety levels for children around playing the sport. The idea of this being a ‘low-stress’ environment was somewhat supported through the quantitative item ‘level of anxiety in relation to playing Auskick’, which demonstrated a trend towards significance, with a reduction in anxiety ratings (with a moderate effect). Furthermore, parents of the children who completed the community program indicated (on average) their children experienced only ‘a little’ anxiety around the program following participation. Providing children with more controlled environment to explore sports and OPA programs generally, may allow them to gain an understanding of the various sensory elements which comprise the ground, giving them an opportunity to adjust to the unfamiliar environment before going on to play the sport within their communities. The low-stress atmosphere of this environment reported in the qualitative feedback is important for several reasons. Firstly, anxiety has been reported as a barrier to participation [22]. Furthermore, higher rates of anxiety have been recorded in both CP [54] and ASD [12], with evidence from a systematic review finding a co-morbid anxiety disorder in approximately 40% of young people with ASD [55]. Given this, strategies that help reduce anxiety levels are imperative to support sport participation for children with disabilities.

### 4.2. The Usefulness of the ‘Pre-Learn’ Program

Results from the post-survey indicate a high number of parents rated the day as very useful or higher. A high number of parents also rated the day as very useful or higher for their child. These ratings mirror qualitative comments which expressed a need for these types of programs as a precursor to participation. In this way, children are provided with an idea of what activities comprise the program, and parents can see how their child responds, giving them a ‘taste’ before signing up. Whilst many parents reported their positive experiences surrounding the ‘Pre-Learn’ program, not all did. Importantly, attendance on the day allowed parents to ascertain their child’s interest in Australian rules football as a sport, allowing parents of those interested to either sign-up their child to the appropriate club within their community or rule out future participation before paying to enroll in a season. This can have benefits from both a time and financial perspective, whereby parents can ascertain whether playing community Auskick is worth both the time and financial commitment. This can have implications for broader PA given the principles of ‘Pre-Learn’ style can be used to assess child interests across a range of sporting and OPA programs.

Thematic analyses indicated several parents conducted further research into the sport, looking for local clubs to enroll their child in following attendance at a ‘Pre-Learn’ day. This behaviour is indicative of the preparation stage of the “stages of change” or transtheoretical model described by Prochaska, et al. [56] (first described in Prochaska and DiClemente [57]). This model postulates that people move through six stages of change, specifically pre-contemplation, contemplation, preparation, action, maintenance and relapse, and has been previously used as a ‘theoretical foundation’ for the acquisition of behaviours such as PA [58] and has been applied to children with disabilities within PA settings [21]. As per this model, several parents became intent on acting to ensure their child had an opportunity to join a season within their community. Several parents reported the excitement their children expressed around joining the community sport following participation at a ‘Pre-Learn’ day.

While the ‘Pre-Learn’ program provides an avenue to reduce some of the barriers to participation in OPA programs it is important to note several parents still highlighted barriers to participation during the ‘Pre-Learn’ program and later when signing up to sport within their community. For example, a child’s elevated anxiety prevented participation at the ‘Pre-Learn’ day, with the family highlighting that they now know their child does not enjoy the sport. This underscores the idea that whilst some children did not enjoy exposure to this program, it still provided an opportunity to assess whether the program was a good fit for their child before progressing to a paid season. However, given children still experienced barriers to participation on the day, larger scale studies with more in-depth qualitative techniques would aid in clarifying the needs of this group and to refine current and develop further strategies to facilitate inclusion. Regarding the broader community program, parents reported barriers around age (a 10-year-old boy felt he was too old for Auskick). Furthermore, despite the potential benefits of a single-day program in terms of time constraints, parents still reported barriers in the community program around finding appropriate session times when their child is not tired (a barrier reported in previous literature [20]), and environmental barriers such as the number of children in each session and the session length (with the latter again reported in previous literature [18]). This is consistent with previous research examining a goal-directed, family-oriented program aimed at improving PA participation outcomes, which noted environmental factors, e.g., time and access to services/equipment as being the main barrier to goals around participation in their local community [59].

#### Attendance in a Program within the Community

Data from the follow-up survey indicated that a small percentage (four [31%] of the 13 parents who responded, 22% of those who initially attended ‘Pre-Learn’) moved through to the action stage [57] and progressed to enrolling their child in the program within the community. Data from this survey also indicated these families maintained their enrolment through the season, with their child completing the full season. The qualitative feedback at this time point further highlighted the ‘usefulness’ of the day, with attendance influencing the decision to enroll their child when they previously may not have considered doing so. Overall these results suggest ‘Pre-Learn’ day programs may be a useful tool in reducing some barriers to sport participation, increasing later participation in sport. This is particularly important given parent ratings at the follow-up time point indicated, on average, the children who participated in a full season enjoyed participation, and as mentioned, experienced only ‘a little’ anxiety in relation to the sport.

### 4.3. Limitations and Recommendations for Future Research

Whilst the current study provides some evidence for the feasibility, specifically ‘acceptability’ of the ‘Pre-Learn’ program, there are several limitations to both the quantitative and qualitative components of this research. Firstly, the lack of a robust, validated measure and the small number of participants limited all conclusions that can be drawn from quantitative analyses. It is important to note that low statistical power can also overestimate effect sizes [60], hence all results and estimated effects must be interpreted with caution. The limitations around sample size also impacted the level of analysis that could be completed, for example, the impact of age on results was not able to be either controlled for or assessed. Given age was discussed as an influencing factor for some families, future research should aim to consider this further. Larger scale studies using valid and reliable measures are needed to produce more robust quantitative results and assess any differences across diagnostic and age-related groups. Larger scale studies with robust measures would also allow for an evaluation of the potential benefits of the program within the community, following participation across a whole season. Furthermore, 11 children had previously participated in OPA programs prior to attending the ‘Pre-Learn’ program. However, of that number, only four children had participated in football related programs (e.g., soccer or related football (*n* = 2 children with ASD; *n* = 1 with CP-GMFCS III) and wheelchair basketball (*n* = 1 child with CP-GMFCS III). For those children, participation in this program may have been less daunting given their previous exposure to related settings.

Several limitations also lie in the techniques used to collect qualitative data. Only short online survey responses were available for qualitative analyses. An interview (either face-to-face or telephone) would have allowed researchers to probe participants’ answers to open-ended questions and achieve more in-depth responses. The employment of more rigorous techniques would also have increased the rigour of qualitative coding, for example through the use of a second independent coder. Future research into this program may benefit from also conducting interviews or focus groups with parents, children, and coaches. This may increase the chance of gaining richer insight into participant experiences.

## 5. Conclusions

In summary, the current study provides some evidence of the acceptability of a ‘Pre-Learn’ program for children aged 5–11 years with disabilities. Results indicated the program can be useful in reducing some of the barriers that exist to physical activity participation, participation in a popular Australian sport for children with disabilities. Qualitative feedback indicated the program was generally useful and enjoyable for parents and their children. Moreover, while some barriers to participation on the day itself and to the broader program do exist, for some families, participation in this day initiated a contemplation around future participation leading to enrolment in the sport within the community. While these findings are positive, they require replication with a larger sample and with more robust measures and in-depth qualitative methods. Despite limitations, ‘Pre-Learn’ programs have the potential to reduce some of the barriers to sport and OPA participation for children with disabilities and their parents, consequently promoting inclusion in sporting programs. This would, in turn, provide an avenue for children with disabilities to experience the benefits of sport participation, such as motor and social skill development.

## Figures and Tables

**Table 1:** Demographic information for child participants included in analyses.

	Diagnosis				
	Overall *n* = 15	ASD ^1^ *n* = 10	CP ^1^ *n* = 3	DS *n* = 1	ASD & CP *n* = 1
Age *M* (*SD*)	8.16 (2.23)	7.45 (1.98)	10.01 (1.94)	11.22	6.34
Gender					
Males *n* (%)	12 (80)	8 (80)	2 (66.67)	1 (100)	1 (100)
Females *n* (%)	3 (20)	2 (20)	1 (33.33)	0 (0)	0 (0)
Verbal Communication *n* (%)	15 (100)	10 (100)	3 (100)	1 (100)	1 (100)
Additional Support ^1^ *n* (%)	9 (60.00)	6 (60.00)	2 (66.67)	0 (0)	1 (100)
Additional Diagnoses *n* (%)					
ADHD	2 (13.33)	2 (20)	0 (0)	0 (0)	0 (0)
Intellectual Disability	3 (20)	2 (20)	0 (0)	1 (100)	0 (0)
Problems with Vision	3 (20)	2 (20)	1 (33.33)	0 (0)	0 (0)
Language Disorder	3 (20)	3 (30)	0 (0)	0 (0)	0 (0)
Depression/Anxiety	5 (33.33)	3 (30)	1 (33.33)	0 (0)	1 (100)
Sensory Processing Disorder	2 (13.33)	2 (20)	0 (0)	0 (0)	0 (0)
Oppositional Defiance Disorder	1 (6.67)	1 (10)	0 (0)	0 (0)	0 (0)
Motor Problems *	1 (6.67)	1 (10)	0 (0)	0 (0)	0 (0)
Executive Functioning Disorder	1 (6.67)	1 (10)	0 (0)	0 (0)	0 (0)
Asthma	1 (6.67)	0 (0)	1 (33.33)	0 (0)	0 (0)
Therapy *n* (%)					
Speech Therapy	8 (53.33)	6 (60)	0 (0)	1 (100)	1 (100)
Physical/Physiotherapy	4 (26.67)	0 (0)	3 (100)	0 (0)	1 (100)
Psychology	2 (13.33)	2 (20)	0 (0)	0 (0)	0 (0)
Occupational Therapy	11 (73.33)	8 (80)	2 (66.67)	0 (0)	1 (100)
ABA Therapy ^1^	1 (6.67)	1 (10)	0 (0)	0 (0)	0 (0)
Previous Organised Sport Participation *n* (%)	11 (73.33)	6 (60)	3 (100)	1 (100)	1 (100)
Swimming	7 (46.67)	4 (40)	2 (66.67)	0 (0)	1 (100)
Gymnastics	3 (20)	3 (30)	0 (0)	0 (0)	0 (0)
Dance	2 (13.33)	0 (0)	1 (33.33)	1 (100)	0 (0)
Wheelchair Basketball	1 (6.67)	0 (0)	1 (33.33)	0 (0)	0 (0)
Tennis	1 (6.67)	0 (0)	1 (33.33)	0 (0)	0 (0)
Football (e.g., soccer) ^1^	3 (20)	2 (20)	1 (33.33)	0 (0)	0 (0)

^1^ Additional support = support received in school (i.e., from a teaching assistant or integration aid/personalised curriculum), ASD = autism spectrum disorder, CP = cerebral palsy, DS = Down syndrome, ADHD = attention deficit hyperactivity disorder, ABA = applied behaviour analysis, Previous football experience must not include the Auskick program, * motor problems in this context are not inclusive of CP diagnoses.

**Table 2:** Examples of scenarios presented at each session with CHANGE IT (coaching, how to score, area, number of players, game rules, equipment, inclusion and time).

Training Session	Scenario	Change It Examples
First (physical disabilities)	“A child who uses a mobility aid (walker) wants to participate”	C: Change attitude and think about different ways the child could participate
		H: Make each goal worth more points for the child, encourage others to share the ball with them
		A: Use ‘zones’ to ensure there is more space for the child to execute the skills
		N: Change the number of players on each team
		G: Change the rules e.g., child with a walker could kick the ball off a tee or cone
		E: Change the ball e.g., use a soccer ball which may be easier to kick off a cone
		I: Think of other ways for children to participate e.g., a child may prefer to score
		T: Slow each activity down to give more time for skill execution
Second (neurodevelopmental disabilities)	“A child becomes upset and begins to cry during the transition from one activity to another”	C: Change transitioning from activities (implement a visual schedule so children know what activity is coming up/when session is ending)
		H: Don’t penalize teams s for those who are hesitant to take part
		A: Ensure clear area for each activity for all children to see
		N: Create groups of children who happily transitioned but ensure it is flexible for others to rejoin
		G: Child may not want to join in new activity right away, allow for breaks so they can join back in when ready
		E: Make use of a quiet area for children to go
		I: Think of other ways for children to participate, e.g., child may prefer to score
		T: Provide clear warnings before the activity changes to allow time for children to prepare

**Table 3:** Likert-scale items used in online surveys.

Item Number	Item Description
1	Task skill and performance (e.g., footy skills like kicking and running)
2	Level of independence performing tasks required in Auskick
3	Confidence in ability to perform the tasks required in Auskick
4	General self-confidence
5	Feelings of satisfaction and pride in playing Auskick
6	Enjoyment of Auskick
7	Preference of Auskick over other organised physical activities
8	Preference for Auskick over other activities (not just physical activities)
9	Level of anxiety in relation to playing Auskick

**Table 4:** Open-ended questions used in survey and qualitative response rates to these questions.

Open-Ended Questions	Response Rate
Post Survey (*n* = 15)	
What would you change about the ‘AllPlay Pre-Learn’ Day?	93% (no feedback from *n* = 1 child with ASD)
What would you not change about the ‘AllPlay Pre-Learn’ Day?	87% (no feedback from *n* = 1 child with ASD and *n* = 1 child with CP)
Would you recommend the ‘AllPlay Pre-Learn’ Day to families of children with additional needs who may be thinking about enrolling in Auskick? Tell us why or why not	80% (no feedback from *n* = 2 children with ASD and *n* = 1 child with DS)
Have you enrolled or are you planning to enrol your child in Auskick this season? Feel free to tell us more if you wish.	93% (no feedback from *n* = 1 child with ASD)
**Follow-up Survey (*n* = 13)**	
Did you enrol your child in Auskick this season? Feel free to tell us why or why not if you wish.	54% (no feedback from *n* = 3 children with ASD, *n* = 2 with CP, *n* = 1 with DS)
Did your child complete the 2018 Auskick season? Feel free to tell us why or why not if you wish.	54% (no feedback from *n* = 3 children with ASD, *n* = 2 with CP, *n* = 1 with DS)

**Table 5:** Median Pre and Post ‘Pre-Learn’ Day Parent Survey Scores and Accompanying Test Statistics (*n* = 14).

Item Description	Pre	Post	*T*	*Z*	*p* *	*r* ^a^
	*Mdn*	*Mdn*				
Task skill and performance	2.00	2.00	12.00	−1.31	0.29	−0.25
Independence in performing Auskick tasks	2.00	2.50	6.00	−1.41	0.25	−0.27
Confidence in ability to perform Auskick tasks	2.00	2.50	10.50	−1.10	0.34	−0.21
General self-confidence	2.00	3.00	4.00	−1.89	0.13	−0.36
Satisfaction and pride playing Auskick	3.00	4.00	2.00	−1.52	0.25	−0.29
Enjoyment of Auskick (*n* = 13)	3.00	4.00	8.00	−1.81	0.11	−0.36
Preference for Auskick over other OPAs	3.00	3.00	13.50	−0.65	0.63	−0.12
Preference for Auskick over other OAs	3.00	2.50	22.50	0.00	1.00	0.00
Level of anxiety in relation to playing Auskick	3.00	2.00	8.00	−2.07	0.06	−0.39

* *p*-values reflect exact significance (two-tailed), OPAs = organised physical activities, OAs = organised activities, ^a^ = effect size calculated via the formula $\frac{Z}{\surd n}$.

**Table 6:** Parent-ratings of variables for participants who completed the community football season.

Item Description	*Mdn*	*Min*	*Max*
Task skill and performance	3.00	2.00	5.00
Independence in performing Auskick tasks	2.50	2.00	5.00
Confidence in ability to perform Auskick tasks	3.00	2.00	5.00
General self-confidence	4.00	3.00	5.00
Satisfaction and pride playing Auskick	4.00	4.00	5.00
Enjoyment of Auskick (*n* = 3)	4.00	4.00	5.00
Preference for Auskick over other OPAs	3.50	3.00	5.00
Preference for Auskick over other OAs (*n* = 3)	4.00	3.00	5.00
Level of anxiety in relation to playing Auskick	2.00	1.00	3.00
